# Dr. Morton on Protracted Lactation, &c.

**Published:** 1832-01-01

**Authors:** 


### REMARKS ON LACTATION; WITH OBSERVATIONS ON THE HEALTHY AND DISEASED CONDITIONS OF THE BREAST-MILK, &c. &c. By *Edward Morton*, M.D. Cantab. Octavo, October, 1831.

SOME part of this work was published four years ago in a contemporary journal, and we are informed by the author that subsequent experience in infantile diseases has convinced him of the correctness of the views which he then broached. The volume is divided into three chapters, the first of which contains nothing that need detain us here. The second is on the disorders that are frequently produced by lactation, especially protracted lactation.

"In cases of extreme delicacy of constitution, lactation will often produce the worst effects. Many young ladies, on becoming mothers, are incapable of supporting the constant drain to which the wants of their infants subject them—they lose their good looks, become gradually weaker, and as their strength declines, their milk is simultaneously lessened in quantity, and altered in its other properties.

If the suckling be still continued, their debility daily increases, distressing pains in the back and loins succeed ; the patients become exceedingly nervous, as it is termed, and are unusually susceptible of ordinary impressions ; pain in the head, often of great violence, follows, which, in some cases, is succeeded by delirium, in others, by absolute mania. Nor is this the whole catalogue of ills to which in such cases the unfortunate mother is subjected : the appetite fails, distressing languor is experienced by day, while copious perspirations deluge her by night, and dissipate the last remains of strength—producing a state which may easily be mistaken for, or terminate in, true pulmonary consumption ;—finally, the sight becomes progressively weaker, until vision is almost destroyed ; the eyelids exude a glutinous secretion, and ophthalmia itself is occasionally induced.

These are the symptoms too often caused by lactation in delicate or debilitated habits, even a few months after delivery; the same also are observed when suckling has been injudiciously protracted beyond the period to which it should be confined.

A few only of the foregoing symptoms may be noticed, or nearly the whole may present themselves, in the same patient; and when this happens, unless the cause which has given rise to them be at once detected, and appropriate treatment employed, the most serious consequences may be apprehended." 17.

In such cases, Dr. M. observes, half measures are useless, or worse. Suckling must be discontinued altogether, for reasons which the author states, and which are sufficiently cogent. The obvious dependency of the foregoing symptoms on debility, will lead the practitioner to the employment of tonics and other means of invigorating the constitution. Injudicious practitioners have sometimes recourse to cupping for the head-ache, which of course aggravates the evil. The occurrence of miscarriage from suckling is illustrated by a case in private practice; and the author says that it is made public at the desire of the patient herself. We shall quote this case.

" Mrs. A——, a lady of delicate constitution, about twenty years of age, three or four months subsequent to the birth of her first child, began to find her milk gradually lessen in quantity; it had also much changed from its previous appearance, resembling at the time just stated, a yellowish, turbid serum. Her child became emaciated; and diarrhœa supervening, my professional services were required. My advice was, that the child should be at once weaned, and a suitable wet-nurse, if possible, procured—neither of which suggestions, as will shortly appear, were followed. I urged the necessity of this measure more particularly, because Mrs. A. was daily getting thinner and weaker; she also complained of great pain in the head and back, and of an increasing dimness of sight, which made her fear she should become blind; but the mother-in-law of my patient being, unfortunately, of opinion that pregnancy in the latter would not again occur during the continuance of lactation, recommended that the child, although chiefly supported upon spoon-meat, should occasionally be allowed to take the breast; and this plan, notwithstanding the wish of Mrs. A—— to the contrary, and my own remonstrances on the subject, was adopted—the effects of which were to increase the mother's ailments, as well as those of her infant. Things went on thus for some time longer, when I once more endeavoured to persuade Mrs. A—— to follow my advice, observing, that by an opposite line of conduct she was not only injuring her own health, but that of her child, neither of which, I assured her, in my opinion, would be re-established till the latter had been weaned. I expressed also my complete incredulity as to the non-recurrence of pregnancy in consequence of her infant remaining at the breast; and I added—' It is my firm conviction that if you be pregnant, or should happen shortly to become so, you will miscarry.' About a week after this conversation she was suddenly seized with flooding, and what I had predicted took place. She now left off suckling, and in about a month, under suitable treatment, completely got rid of all her former complaints; the child also immediately began to improve." 22.

The third chapter embraces the subject of diseases in children, resulting from protracted lactation. To this source our author traces cases of vomiting, diarrhœa, debility, scrofula, tabes, convulsions, epilepsy—and, lastly, meningitis, with hydrencephalus. This is a long and frightful list of maladies sucked from the breasts of our mothers! It is to meningitis, however, that Dr. Morton chiefly directs his attention. The conclusions to which our author has come, from experience, are as follow:

"1st,—That if children be suckled for an undue length of time, they will be liable in consequence to be affected with meningitis, or inflammation of the investing membranes of the brain.

2dly,—That should they not become affected with the disorder in question during or soon after the time they are thus improperly suckled, they will nevertheless acquire therefrom a predisposition to cephalic disease at some future period of their lives.

3dly. That children who are suckled for an undue length of time, when labouring under other diseases, will be much more liable to have the head secondarily affected, than children brought up in a different manner.

4thly,—and lastly, that the same effects will take place in infants if suckled by women who have been delivered an undue length of time; although the infants themselves may not have been at the breast for too long a period." 26.

The author then proceeds to give an abstract of 52 cases, illustrating the foregoing views. Among these are five cases communicated by Mr. Griffith, an intelligent surgeon in Pimlico. For these histories we must refer to the work itself. With respect to the manner in which protracted lactation causes the complaint, Dr. Morton has no doubt that the meningitis is a secondary affection, arising from the deranged function of the digestive organs—that derangement itself originating in the depraved condition of the breast-milk. Dr. Morton supports this opinion by the authority of other writers, and of Mr. Dendy in particular. The following passage is from the latter writer.

"It may be truly said, that *the infantine disease excited by milk of a deleterious, or simply impoverished quality*, '*grows by what it feeds on;*' and we shall witness the internal debility and the infantine disorder running their course together. Tabes is the natural consequence of this error; but its effect is evinced by the occurrence of other disorders. A defective degree of nutrition, as I have elsewhere stated, predisposes the system to become influenced by comparatively slight excitement; and thus, in addition to the direct excitement of disease, it becomes indirectly its predisposing cause. *Under its influence the serous* and mucous *membranes become readily the seat of inflammatory action.*" 42.

The author concludes his work with an invitation to practitioners, to convey to him such information as they may possess on this interesting subject, in order that he may avail himself of it in a new edition. We think Dr. Morton has adduced sufficient facts and reasons for drawing the attention of practitioners to the diseases resulting from protracted lactation.

---

